# Therapeutic effects of myrrh extract and myrrh-based silver nanoparticles on *Trichinella spiralis*-infected mice: parasitological, histopathological, and immunological (IFN-γ, IL-10, and MMP-9) investigations

**DOI:** 10.3389/fvets.2024.1433964

**Published:** 2024-10-03

**Authors:** Salwa Mahmoud Abd-ELrahman, Ahmed Kamal Dyab, Abeer El-sayed Mahmoud, Shaymaa M. Mohamed, Alamira Marzouk Fouad, Ahmed Gareh, Jamal Asseri, Naief Dahran, Hind Alzaylaee, Hayat M. Albisihi, Ahmed Mahmoud Abd Elrahman, Fahd M. Alsharif, Heba Mostafa, Nashwa Hamad, Ehab Kotb Elmahallawy, Nahed Ahmed Elossily

**Affiliations:** ^1^Department of Parasitology, Faculty of Veterinary Medicine, Assiut University, Assiut, Egypt; ^2^Department of Medical Parasitology, Faculty of Medicine, Assiut University, Assiut, Egypt; ^3^Department of Parasitology, School of Veterinary Medicine, Badr University in Assiut, Assiut, Egypt; ^4^Department of Pharmacognosy, Faculty of Pharmacy, Assiut University, Assiut, Egypt; ^5^Department of Aquatic Animal Medicine and Management, Faculty of Veterinary Medicine, Assiut University, Assiut, Egypt; ^6^Department of Parasitology, Faculty of Veterinary Medicine, Aswan University, Aswan, Egypt; ^7^Department of Biology, College of Science and Humanities, Shaqra University, Dawadmi, Saudi Arabia; ^8^Department of Anatomy, Faculty of Medicine, University of Jeddah, Jeddah, Saudi Arabia; ^9^Department of Biology, College of Science, Princess Nourah bint Abdulrahman University, Riyadh, Saudi Arabia; ^10^Department of Biochemistry, College of Science, King Abdulaziz University, Jeddah, Saudi Arabia; ^11^Department of Chemistry, Faculty of Science, Assiut University, Asyut, Egypt; ^12^Department of Pharmaceutics and Industrial Pharmacy, College of Pharmacy, Al-Azhar University, Assiut, Egypt; ^13^Department of Anatomy and Embryology, Faculty of Veterinary Medicine, Assiut University, Assiut, Egypt; ^14^Department of Pathology, Faculty of Veterinary Medicine, Assiut University, Assiut, Egypt; ^15^Departamento de Sanidad Animal, Grupo de Investigación en Sanidad Animal y Zoonosis (GISAZ), UIC Zoonosis y Enfermedades Emergentes ENZOEM, Universidad de Córdoba, Córdoba, Spain; ^16^Department of Zoonoses, Faculty of Veterinary Medicine, Sohag University, Sohag, Egypt

**Keywords:** *Trichinella*, cytokine, silver nanoparticles, myrrh, anthelmintic, histopathology, immunology

## Abstract

**Introduction:**

Trichinellosis, caused by *Trichinella spiralis* (*T. spiralis*), remains a prevalent parasitic zoonosis. Developing new drugs targeting and understanding the immune response against the infection is imperative. Previous research has inadequately explored the efficacy of crude myrrh extract and myrrh-based silver nanoparticles (AgNPs) against trichinellosis, as well as their impact on histopathological, and immunological factors.

**Methods:**

This study evaluated the effects of silver nanoparticles biosynthesized using myrrh, crude myrrh extracts, and albendazole on the intestinal phase of *T. spiralis*. It also examined the associated histopathological changes and alterations in key immunological markers, including Interferon-gamma (IFN-γ), Interleukin-10 (IL-10), and Matrix Metalloproteinase-9 (MMP-9). Five groups of 12 mice were allocated as follows: group 1: non-infected, non-treated (negative control), group 2: infected, non-treated (positive control), group 3: infected and treated with biosynthesized silver nanoparticles (40 μg/mL), group 4: infected and treated with myrrh crude extract (800 mg/kg), and group 5: infected and treated with albendazole (50 mg/kg). Treatment was orally administered starting on the 2^nd^ day post-infection and continued for three successive days. Mice of all groups were euthanized on the 6^th^ day post-infection, and the intestine of each was isolated for parasitological, histopathological, and immunohistochemistry evaluation of MMP-9, as well as assessment of cytokines level (IFN-γ and IL-10 gene expressions) via Real-time PCR technique.

**Results:**

The present study showed a considerable reduction in adult worm count among the treated groups. The mortality rates of adult worms were 88.64% in the silver nanoparticles treated group, 85.17% in the myrrh crude extract group, and 94.07% in the albendazole-treated group. Histopathological examination revealed prominent alterations in the intestine of the infected non-treated mice, which were markedly restored by treatment. Immunohistochemical examination accompanied by significant reduction in MMP-9 expression in the infected mice treated with AgNPs compared to the infected non-treated group, reflecting the role of AgNPs in downgrading the inflammatory reaction in the intestine of infected mice.

**Conclusion:**

Collectively, this study demonstrates the novel antiparasitic potential of silver nanoparticles biosynthesized with myrrh against *T. spiralis* in infected mice. The treatment was associated with moderate rise in IFN-γ gene expression and IL-10 expression, highlighting its therapeutic efficacy against *T. spiralis*.

## 1 Introduction

Trichinellosis, a globally prevalent zoonotic illness, arises from infection with the nematode parasite *Trichinella*, notably *T. spiralis*. This parasite infects a wide array of mammalian hosts, including humans, who mainly acquire the infection through the ingestion of raw or undercooked pork containing larvae (1, 2). Importantly, *T. spiralis* exhibits a unique ability to infect various hosts due to its lifecycle stages coexisting within a single host (3). The lifecycle of *T. spiralis* encompasses two key phases: the intestinal and muscular stages. Disease progression heavily relies on the host’s immune response, which is crucial for expelling adult worms from the small intestine. Early infection triggers a multifaceted immune process involving different immune cell types and cytokines (4). Notably, cytokines such as IFN-γ and IL-10 play pivotal roles in the immune response against *T. spiralis*. Upon T cell stimulation, activated macrophages produce IFN-γ, which demonstrates robust parasite-killing properties (5, 6). Moreover, IL-10 serves as an anti-inflammatory mediator, dampening pro-inflammatory cytokine release and preventing liver necrosis during acute trichinellosis (7). Meanwhile, IFN-γ enhances the cytotoxicity of various immune cells against *T. spiralis* larvae (6). The host mounts an innate and adaptive IL-10 response to mitigate tissue damage and immune-mediated inflammation, aiding *T. spiralis* intracellular infection (5). The efficacy of the immune response is associated with regulatory cytokines like IL-10 and Transforming growth factor beta (TGF-β), which counterbalance pro-inflammatory cytokines such as IFN-γ, IL-6, and IL-17 (8). Differential expression patterns of cytokines like IFN-γ and IL-12 occur during different stages of infection, highlighting the dynamic immune response during trichinellosis (4, 9). Furthermore, Matrix metalloproteinases (MMPs) are a group of structurally related proteins that play a collective role in the metabolism of the extracellular matrix (ECM) of the connective tissue. They are produced by various cells including epithelial and endothelial cells, fibroblasts, platelets, neutrophils, eosinophils, and macrophages (10). These endopeptidases are responsible for degrading most ECM components, and therefore, they are involved in the remodeling and degradation of matrix components, such as collagen, proteoglycans, and glycoproteins (11). Additionally, MMPs play a crucial role in inflammatory processes (12, 13). Among them, MMP-9, a 92 kDa gelatinase, plays a vital role in inflammation, tissue healing, and remodeling (14). MMP-9 levels increase during the intestinal phase of *T. spiralis* infection, reflecting its involvement in the inflammatory response (12, 15).

Albendazole is the mainstay treatment for trichinellosis and has marked impact on the immune profile during parasitic infections (16), yet its effectiveness against encapsulated larvae is restricted, raising concerns about drug resistance. Hence, exploring alternative anti-parasitic compounds, particularly from natural sources, is imperative (3). Nanotechnology combined with plant extracts offers a novel approach to parasitic disease treatment, leveraging the efficacy and affordability of natural remedies (17). Myrrh, derived from *Commiphora* species, possesses various medicinal properties, including analgesic, antibacterial, and antifungal effects (18). Taken into consideration, myrrh has a property to combat the cell damage through down regulation of TNF-α and IL-6 (inflammatory cytokines) and this led to reduction of the hepatic injury. Notably, myrrh can mitigate cellular damage by modulating inflammatory cytokines like TNF-α and IL-6, indicating its potential therapeutic value (19). Limited research has explored the *in vivo* activity of myrrh crude extract and myrrh-based silver nanoparticles against both intestinal and muscular stages of *T. spiralis* (20, 21), and their effects on the immune profile remain unexplored. Thus, this study aimed to evaluate the efficacy of myrrh extract and myrrh-based silver nanoparticles against the intestinal stage of *T. spiralis* infection in mice. Furthermore, it investigated the immunological responses, focusing on IFN-γ, IL-10, and MMP-9, while reporting histopathological and immunohistochemical findings, with potential implications for combatting this zoonotic disease.

## 2 Materials and methods

### 2.1 Ethical consideration

The work was approved (Approval Number: 06/2022/0015) by the research ethical committee of the Faculty of Veterinary Medicine, Assiut University.

### 2.2 Infection of mice with *Trichinella spiralis*

In this step, *T. spiralis* strains were maintained through successive *in vivo* passages in BALB/c mice at the animal facility of Assiut University in Egypt, maintained under specific pathogen-free conditions. The mice were fed a standard diet and provided with water while being housed in suitable conditions. Larvae were isolated from the skeletal muscle of infected mice via digestion, with approximately 350 larvae used to infect healthy mice in subsequent passages (21).

### 2.3 Preparation of methanolic myrrh extract and silver nanoparticles (AgNPs) using myrrh extracts

#### 2.3.1 Preparation of methanolic myrrh extract

Myrrh materials were procured from an herbal market in Assiut, Egypt. The exudate was sourced from the stems of *Commiphora myrrha*, belonging to the *Burseraceae* family. Verification of the exudate’s identity was conducted through the analysis of its physical properties and standard chemical testing procedures, as detailed elsewhere (22). A quantity of 125 g of powdered myrrh material was soaked in 1 L of methanol for 24 h. The following day, the soaked material underwent ultrasonic treatment for 1 h in a sonicator bath (Crest Ultrasonics CP2600HT Ultrasonic Cleaner, India Pvt. Ltd.) to expedite extraction. The resulting extract was then filtered, and the extraction process was repeated five times. Each time, the filtrate was concentrated under vacuum at 40°C using a rotary evaporator. The dried residues obtained from each extraction were combined to yield a total crude extract weighing 28 g.

#### 2.3.2 Green biosynthetic approach for silver nanoparticles (AgNPs) using methanolic myrrh extract

Green biosynthetic technique was applied for the preparation of silver nanoparticles (AgNPs, 100 μg/mL) (23). Briefly, a solution of AgNO_3_ (0.95 mM) in 20 mL of distilled water was combined with 20 mL of the prepared myrrh extract and stirred vigorously using a magnetic stirrer for 1 h. The transition in color, progressing from a faint yellow hue to a deep yellow and ultimately to green, signifies the formation of AgNPs (21).

### 2.4 Experimental design

**Figure 1:**
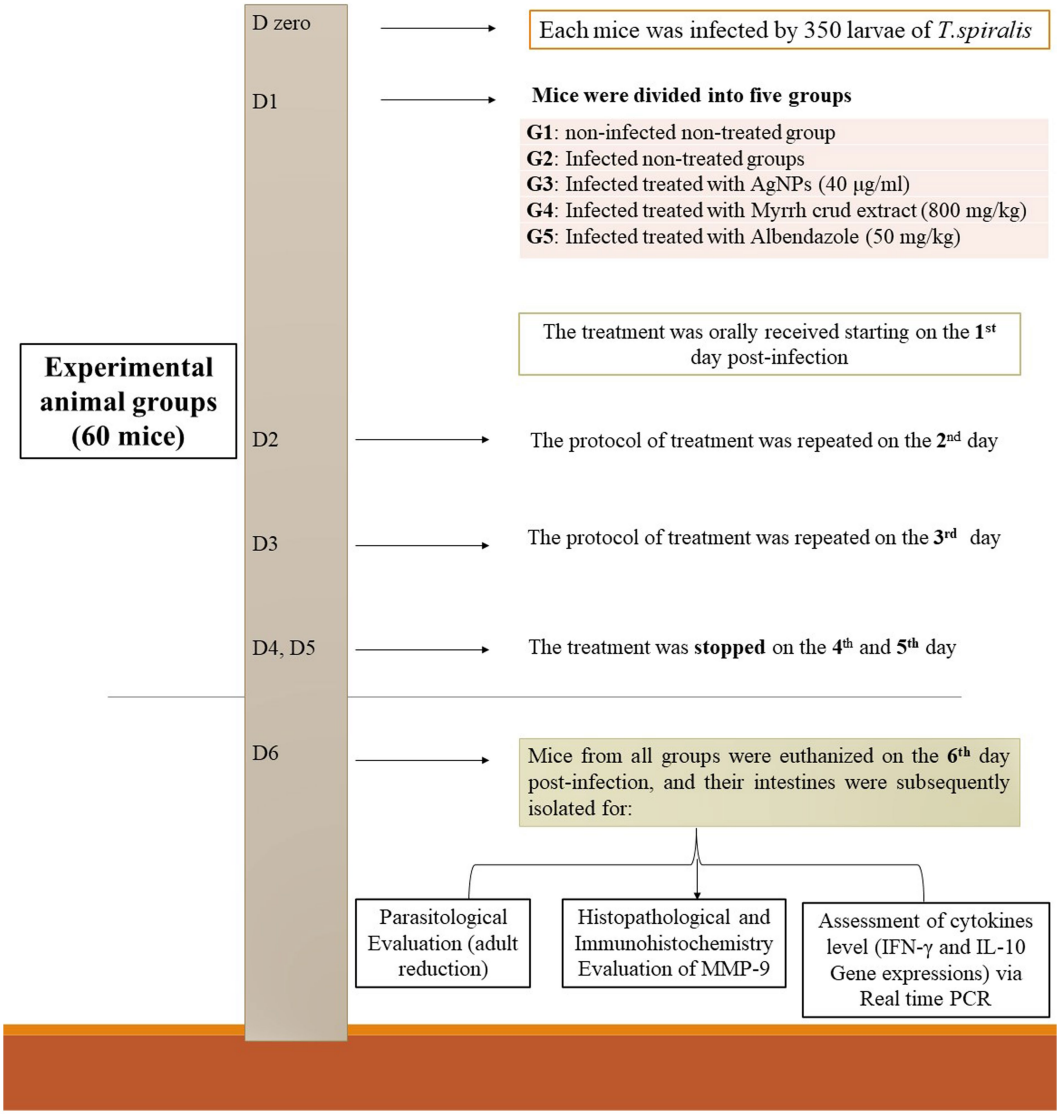
The treatment strategy employed in this study’s experimental protocol involved several steps.

Sixty apparently healthy BALB/c mice, free of parasites, aged 6 weeks and weighing between 20 and 25 g, were included in the study. After acclimatization for 1 week, at age of 7 weeks, they were divided into five groups, each comprising 12 mice as follows: group 1 (G1) served as the non-infected, non-treated (negative control); group 2 (G2) represented the infected, non-treated (positive control); group 3 (G3) received treatment with biosynthesized AgNPs (40 μg/mL); group 4 (G4) received treatment with myrrh crude extract (800 mg/kg); and group 5 (G5) received treatment with the reference drug (50 mg/kg albendazole). Each mouse was orally inoculated with 350 *T. spiralis* larvae. All treatment were orally adminstrated for all groups commenced the day after infection and continued for three consecutive days. On the 6^th^ day post infection, all mice were euthanized, sacrificed and their intestines were isolated for parasitological, histopathological, immunohistochemical, and immunological evaluation of IFN-γ and IL-10 gene expressions using real-time PCR technique (24, 25). The experimental protocol and treatment strategy are depicted in Figure 1.

### 2.5 Evaluation of the effectiveness of the treatment

As described (3, 24, 26), on the sixth day post-infection, mice from each group were euthanized to evaluate the effects of various treatment agents on adult worms during the intestinal phase and to measure different parameters.

#### 2.5.1 Parasitological efficacy

The intestines of euthanized mice were isolated and cleaned by washing with normal saline (0.9%) to remove intestinal contents. Subsequently, the intestines were opened, and the mucosa was gently scraped. The collected adult worms were then incubated in PBS for 4 h at 37°C. After incubation, the worms were collected and counted using a dissecting microscope. The efficacy of treatment was calculated using the following formula: percent of adult reduction (%) = 100 × (Mean number recovered in controls − Mean number recovered in treated mice)/Mean number recovered in control (26).

#### 2.5.2 Histopathological analysis

To assess histopathological changes, specimens from the small intestine of each group were obtained, fixed in 10% neutral buffered formalin, and processed for conventional histopathological examination at the Tissue Processing Laboratory of the Pathology and Clinical Pathology Department, Faculty of Veterinary Medicine, Assiut University. Following fixation, tissue specimens underwent dehydration, embedding in paraffin wax, and sectioning into 5 μm thick slices using a Leica RM2125 microtome (Leica Microsystems, Wetzlar, Germany). These sections were subsequently stained with hematoxylin and eosin (H&E) stain according to Bancroft et al. (27). Evaluation and photography of the sections were performed using a Leitz Dialux 20 microscope and Canon digital camera (Candison Power Shot A 95).

#### 2.5.3 Immunohistochemical analysis

Immunohistochemical analysis was conducted following the method previously described by Taylor et al. (28). Initially, formalin-fixed paraffin-embedded sections from the intestine of all experimental groups underwent de-paraffinization in xylene and rehydration in a descending graded series of ethyl alcohol. Antigen retrieval was then performed in 10 mM sodium citrate buffer (pH 6.0) by boiling the sections in a water bath for 20 min, followed by cooling at room temperature for 20 min. To block nonspecific binding, tissue sections were treated with 5% bovine serum albumin for 1 h. Subsequently, alkaline peroxidase activity in the tissue sections was inhibited with 3% hydrogen peroxide for 10 min at room temperature before incubating with a diluted (1:100) rabbit polyclonal anti-Matrix metallopeptidase 9 (MMP-9) primary antibody (A11147, AB clonal Company, 500W Cummings Park, Ste. 6500, Woburn, MA 01801, United States) overnight in a humid chamber at 4°C. Following overnight incubation, the sections were treated with secondary antibodies at room temperature in a humid chamber for 1 h. After washing, the sections were stained with 3,3′-diaminobenzidine (DAB) chromogen substrate for 10 min and counterstained with Mayer’s hematoxylin. Finally, the sections were dehydrated in an ascending graded series of ethanol, mounted, and examined under a light microscope to visualize brown-colored positive immunoreactions. According to a previous study (29), MMP-9 immunostaining intensity was semi-quantitatively scored by evaluating the intensity of positively stained cells in five high-power fields/slide across six slides, each representing the intestinal sections of six mice in each group. The intensity of MMP-9 staining was graded from 0 to 3 as follows: 0 = none, 1 = weak, 2 = moderate, and 3 = strong. The final scores of all groups were presented as means and subjected to statistical comparison.

#### 2.5.4 Evaluation of selected cytokines by real time PCR-RNA extraction

The total ribonucleic acid (RNA) was extracted from each experimentally infected mouse’s intestinal specimens (jejunal tissue) using the RNeasy mini kit following the manufacturer’s protocol. RNA concentration was determined by measuring optical density at 260 nm and the ratio of optical density at 260/280 nm using a Nanophotometer, and then stored at −80°C. The concentration of RNA was determined by measuring the optical density at 260 nm and ratio optical density at 260/280 nm, using Nanophotometer, and then stored at −80°C.

#### 2.5.5 Reverse transcription

Reverse transcription was performed using Omniscript reverse transcription kits. Complementary deoxyribonucleic acid (cDNA) was synthesized in a 20 μL reaction volume containing omniscript reverse transcriptase (1 μL), 10× RT buffer (2 μL), 1 μM Oligo dT primer, 5 mM dNTP (2 μL), 10 U/μL RNase inhibitor (1 μL), RNA (5 μL), and completed to 20 μL using Diethylpyrocarbonate (DEPC)-treated water. The mixture was incubated at 37°C for 60 min and used for qRT-PCR.

#### 2.5.6 Real-time PCR

The IFN-γ and IL-10 gene expressions were determined using 12.5 micro-liter of 2x SYBR green real-time PCR kit. The reaction mixture (25 μL final volume) contained 2.5 μL of cDNA template, 10 μM of each primer, and sterile PCR-grade water. A no-template PCR control was included. The cycling profile consisted of initial denaturation at 94°C for 5 min, followed by 40 cycles of denaturation at 95°C for 20 s, annealing at 60°C for 25 s, and extension at 72°C for 1 min. A melting curve analysis was performed to confirm the specificity of the PCR products. The housekeeping gene GAPDH was used as an internal control for cDNA normalization.

#### 2.5.7 qPCR data analysis

The value of threshold cycle (CT) was determined using the automatic setting on “Mx3005P real-time QPCR detection system. Delta–delta CT (2^-∆∆CT^) method was used in calculating gene expression of the target genes (30). The target gene sequences, selected according to Ding et al. (4), included IFN-γ with a forward sequence of 5’-CCATCGGCTGACCTAGAGAA-3’ and a reverse sequence of 5’-GATGCAGTGTGTAGCGTTCA-3’, as well as IL-10 with a forward sequence of 5’-CCCTTTGCTATGGTGTCCTT-3’ and a reverse sequence of 5’-TGGTTTCTCTTCCCAAGACC-3’. These sequences were normalized to the GAPDH gene, which served as the housekeeping gene or calibrator, with a forward sequence of 5’-ACCACAGTCCATGAAATCAC-3’ and a reverse sequence of 5’-TCCACCACCCTGTTGCTGTA-3’. The ΔCT (delta CT) values were calculated using the following formulas: ΔCT (required gene) = CT (required gene) - CT (housekeeping gene mean) and ΔCT (control) = CT (control samples) - CT (housekeeping gene mean). Then, the resulted ΔCT of the control samples was subtracted from the delta CT of the treated (target genes) samples and this is the delta–delta CT (CT) as per the formula: ΔΔCT = ΔCT (target gene) − ΔCT (control). The fold change in the target gene expression was obtained by this formula, 2^-∆∆CT^ and the results were subjected to statistical analysis. The threshold cycle (CT) value was determined using the automatic setting on the Mx3005P real-time QPCR detection system. The delta–delta CT (2^-∆∆CT^) method was used to calculate the gene expression of the target genes (30). The results were subjected to statistical analysis.

### 2.6 Statistical analysis

Statistical analysis was performed using the Statistical Package for Social Sciences (SPSS) version 20 for Windows. The data were presented as mean ± standard deviation. Differences between the groups were assessed using one-way ANOVA followed by Duncan’s post-hoc test. A *p*-value less than 0.05 was considered statistically significant.

## 3 Results

### 3.1 Parasitological assessment of different treatments

As illustrated in Table 1, a statistically significant decrease in the count of recovered adult worms was reported across all treated groups compared to the infected non-treated group (*p* < 0.0001). Table 1 also demonstrated a notable reduction in the number of retrieved adult worms, with percent of adult reduction of 88.64 and 85.17% in the groups treated with AgNPs and Myrrh crude extract, respectively. Similarly, the Albendazole-treated group exhibited a percent of reduction of 94.07%.

**Table 1:** Percentages of adult worms’ reduction in the intestinal phase of all infected treated groups, compared to the infected untreated group.

Groups	G2	G3	G4	G5
Dose	Infected untreated	40 μg/mL (biosynthesized AgNPs)	800 mg/kg (myrrh crude extract)	50 mg/kg (albendazole)
Mean ± SD	196.67 ± 9.4	22.33 ± 1.6*	29.17 ± 9.17*	11.67 ± 4.9*
% of reduction	–	88.64	85.17	94.07
*p* value	<0.001			

*p*-value < 0.001. *Statistically significant in all treated group compared to the corresponding value in the infected untreated group.

### 3.2 Histopathological observations

**Figure 2:**
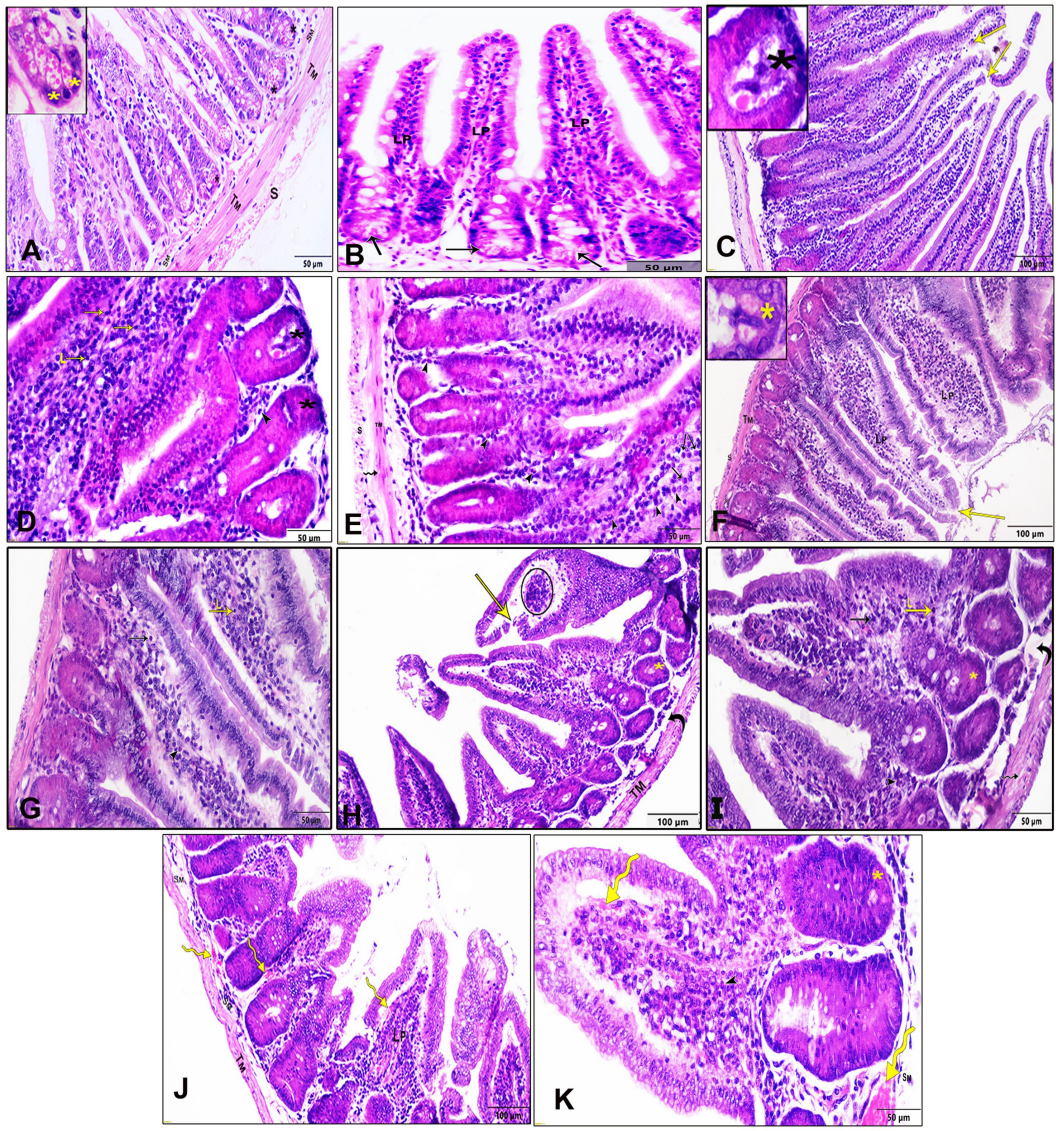
Histopathological examination of the intestinal tissue sections of different study groups stained by H&E stain. **(A,B)** Negative control group (G1), **(C–E)** positive control mice (G2), **(F,G)** Infected mice treated with AgNPs (G3), **(H,I)** infected group received crude extract of myrrh (G4), and **(J,K)** infected group treated with albendazole (G5), lamina propria (Lp), submucosa (SM), tunica muscularis (TM), serosa (S), epithelial necrosis, and shedding (yellow arrows), eosinophils (arrowheads), mast cells (black arrows), and lymphocytes (L), Paneth cells (asterisks), and cytoplasmic vacuolation in the muscle layer (wavy arrow), submucosal edema (curved arrows), vascular congestion (yellow wavy arrows), and necrotic tissue infiltrated with inflammatory cells (circle).

The histopathological assessment of intestinal sections from the negative control group (G1) revealed a normal architecture characterized by four distinct layers: tunica mucosa, tunica submucosa, tunica muscularis, and tunica serosa. The mucosa displayed epithelial villi with few goblet cells and crypts at the base of each villus. Small clusters of Paneth cells with numerous apical cytoplasmic granules and basally located nuclei were observed at the crypt ends (Figures 2A,B). In contrast, intestinal sections infected with *T. spiralis* (G2, non-treated group) exhibited marked necrosis and sloughing of the epithelial covering, accompanied by pronounced inflammatory cellular infiltration in the lamina propria, predominantly eosinophils, mast cells, and lymphocytes. Additionally, vascular congestion, edema, and necrosis of Paneth cells were evident. The muscle layer displayed cytoplasmic vacuolation (Figures 2C–E). Treatment of the infected mice with AgNPs (G3) demonstrated marked improvement in the histological appearance of the intestine with mostly regular villous patterns. Mild epithelial necrosis was observed at the tips of some villi, along with moderate inflammatory cellular infiltration in the lamina propria, including lymphocytes and eosinophils (Figures 2F,G). In the group treated with the crude extract of myrrh (G4), intestinal sections showed evidence of villar epithelial necrosis. Moderate infiltration of inflammatory cells in the lamina propria, comprising lymphocytes, eosinophils, and a few mast cells, was also noted. Additionally, there were instances of Paneth cell necrosis, faint pink edema fluid in the submucosa, and vacuolation of the muscle layer (Figures 2H,I). Similar microscopic alterations were observed in the group treated with albendazole (G5), with notable congestion in the blood vessels of the lamina propria and submucosa (Figures 2J,K).

### 3.3 The immunohistochemical assessment using of MMP-9

**Figure 3:**
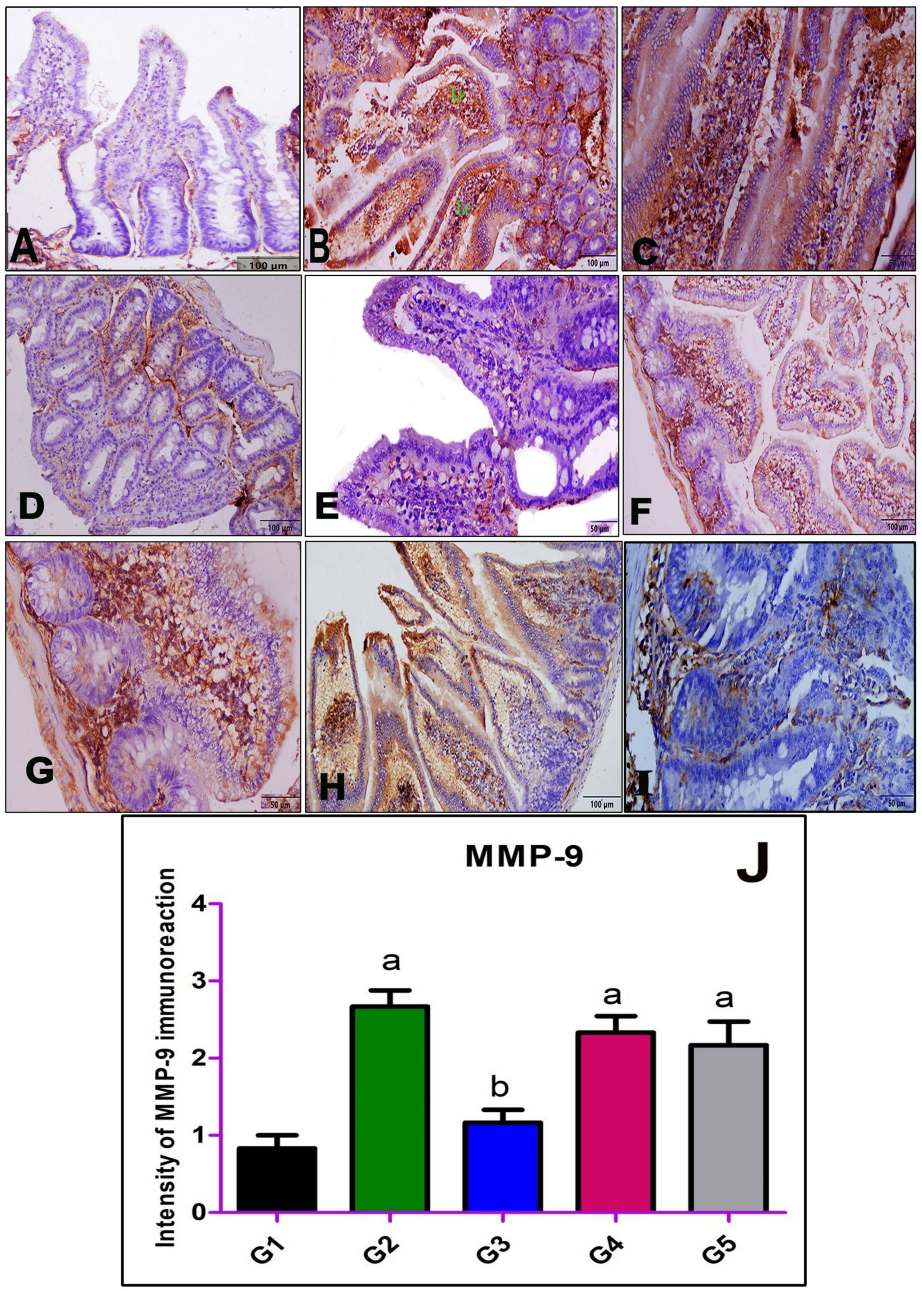
Immunohistochemical staining of MMP-9 in the small intestine; **(A)** negative control group (G1), **(B,C)** positive control mice (G2), **(D,E)** infected mice treated with AgNPs (G3), **(F,G)** infected group received a crude extract of myrrh (G4), and **(H,I)** infected group treated with albendazole (G5) showing weak immunoreactivity of MMP-9 in the normal control group (G1) **(A)**, strong cytoplasmic immunoreactivity in the inflammatory cellular infiltrate within the villous core in G2 **(B,C)**, and weak cytoplasmic staining in G3 **(D,E)**, while both G4 **(F,G)** and G5 **(H,I)** showing moderate cytoplasmic staining of MMP-9. **(J)** Statistical analysis of the intensity of the immunostaining of MMP-9 in the intestinal section of all studied groups. Values are demonstrated as mean ± SEM (*n* = 6). *p* < 0.05 indicates a significant difference. ^a^(*p* < 0.05) Compared to G1. ^b^(*p* < 0.05) compared to G2.

Immunohistochemical staining of intestinal sections from the normal control group (G1) with anti-MMP-9 antibodies revealed weak immunoreactivity (Figure 3A). In contrast, the expression of MMP-9 in the infected non-treated group (G2) exhibited strong cytoplasmic immunoreactivity within the villous core (Figures 3B,C). Sections from the group treated with AgNPs (G3) displayed weak immunoreactivity against MMP-9 in all intestinal layers (Figures 3D,E). Moderate expression of MMP-9 was observed in the intestinal sections of rats treated with myrrh (G4) and albendazole (G5), as depicted in Figures 3F,G,H,I, respectively. A semi-quantitative analysis of the immunoreaction intensity of MMP-9 in the intestinal sections of all experimental groups was performed and revealed a highly significant increase in G2 compared to G1. However, G3 showed a significant decrease in the immunoreaction intensity of MMP-9 compared to G2 (Figure 3J).

### 3.4 Evaluation of cytokines profile by real time PCR

Both IFN-γ and IL-10 gene expression were investigated to elucidate the immune system’s modulation mechanism against *T. spiralis* infection in experimentally infected mice treated with AgNO_3_ prepared with myrrh (G3), the crude extract of myrrh (G4), and albendazole (G5). In this regard, mRNA expression of both cytokines was detected in the jejunal tissue of mice on the 6th day post-infection using qPCR. In the infected non-treated group (G2), the gene expression of IFN-γ was 2.12 ± 0.97. Conversely, in the silver nanoparticles treated group (G3), IFN-γ expression was 1.15 ± 0.96. The myrrh-treated group (G4) exhibited IFN-γ expression of 3.26 ± 1.22. In the albendazole-treated group (G5), IFN-γ production was 0.57 ± 0.40. However, there was no significant difference between the different treatment and control groups, as indicated in Table 2. Regarding the gene expression of IL-10, its expression differed from IFN-γ. In the infected untreated group (G2), IL-10 expression appeared on the 6^th^ day post-infection, with a level of 1.81 ± 0.64. In the AgNO_3_ treated group (G3), IL-10 expression was 1.22 ± 0.61. The myrrh-treated group (G4) showed delayed IL-10 expression (0.92 ± 0.72). In contrast, the albendazole-treated group (G5) exhibited early IL-10 expression on the 6th day post-infection (2.30 ± 1.13), as shown in Table 2.

**Table 2:** The mRNA gene expression of IFN-γ and IL-10 after different treatment at day 6th.

	The gene expression of IFN-γ		The gene expression of IL-10	
G1	1.00 ± 0.00	ab	1.00 ± 0.00	a
G2	2.12 ± 0.97	ab	1.81 ± 0.64	ab
G3	1.15 ± 0.96	ab	1.22 ± 0.61	a
G4	3.26 ± 1.22	b	0.92 ± 0.72	a
G5	0.57 ± 0.40	a	2.30 ± 1.13	b
	0.07		0.031*	

One-way ANOVA was conducted to assess mean differences between groups, with asterisks (*) indicating a significant difference between treatment and control groups *p* < 0.05. Similar letters indicate no significant difference between groups, while different letters denote a statistically significant difference at the 0.05 significance level.

## 4 Discussion

The present study’s findings highlighted the effectiveness of biosynthesized silver nanoparticles and myrrh crude extract in treating the intestinal phase of *T. spiralis*, comparable to Albendazole as a reference drug. This evaluation encompassed parasitological and histopathological effects, as well as the immune response through the assessment of IFN-γ and IL-10 levels. In this work, all treated groups showed a notable decrease in the average adult worm count compared to the positive control group (*p* < 0.001), silver nanoparticles prepared with myrrh are more effective followed by myrrh crude extract. These results aligned with a previous investigation by Elossily et al. (26), which also indicated the beneficial effects of myrrh and myrrh-based silver nanoparticles on the intestinal phase of *T. spiralis*. However, this study observed that silver nanoparticles exhibited greater efficacy than crude myrrh extract, which may be attributed to differences in the timing of treatment initiation. In the current study, treatment commenced on the day following infection, coinciding with the larval-to-adult transformation stage. Based on our previous research indicated that silver nanoparticles combined with myrrh exhibit superior larvicidal effects (21) compared to crude myrrh extract alone (31), we initiated treatment earlier in the infection cycle. This strategy aimed to target the larvae and hinder their development into the adult stage. It should be noted that the infection with *T. spiralis* triggers a cascade of pathological changes in the host’s intestine. An acute inflammatory response manifests as infiltration of mucosal cells by eosinophils, dendritic cells, mast cells, goblet cells, and cytokines, pivotal in shaping the host’s immune response during the early intestinal phase of infection and influencing its outcome (32). In the present study, we investigated the intestinal mucosal immune status during the intestinal phase of *T. spiralis* infection using both histopathological and immunohistochemical analyses. The histopathological examination revealed prominent alterations in the intestines of infected, untreated mice. These changes included epithelial necrosis and shedding, significant infiltration of the lamina propria by eosinophils, mast cells, and lymphocytes, as well as Paneth cell necrosis and vacuolation of the muscular layer. Similar histopathological findings were reported in previous studies conducted by Saracino et al. (33) and Elmehy et al. (34). In this concern, a previous investigation (35) attributed these changes to excretory-secretory products released by adult *T. spiralis*, such as serine and cysteine proteases, which facilitate intestinal penetration and invasion of epithelial cells. In terms of immunohistochemical analyses, our findings revealed an upregulation of MMP-9 expression in the intestinal sections of the infected, untreated group. However, there was a significant reduction in MMP-9 expression in mice treated with AgNPs compared to the infected, untreated group, indicating the potential of AgNPs to mitigate the inflammatory response in the intestines of *T. spiralis*-infected mice. This effect may be attributed to the targeted detrimental effect of AgNPs on adult worms in the intestine, leading to a subsequent decrease in inflammatory cellular infiltration. This observation aligns with the findings of Abd-Elrahman et al. (21), who demonstrated the *in vitro* detrimental impact of AgNPs on *T. spiralis* larvae. Conversely, infected mice treated with a crude extract of myrrh and albendazole exhibited non-significant immunoexpression of MMP-9 compared to the infected, untreated group.

The present study investigated the levels of cytokines (IFN-γ and IL-10) in various treated (AgNPs, myrrh crude extract, and albendazole) and untreated groups infected with *Trichinella*. In the untreated infected group (G2), IFN-γ expression at 6th days post-infection (pi) was 2.12 ± 0.97, and IL-10 levels were measured at 1.81 ± 0.64 on the same day pi. These results come inconsistent with previous studies showing increased mRNA and protein levels of both IFN-γ and IL-10 in *T. spiralis* infected untreated mice (4, 24), expression of proinflammatory and anti-inflammatory cytokines at the beginning of the infection suggests an inadequate immune response. This finding aligns with prior research (4, 24, 36) indicated that the immune system’s inability to combat infection. Moreover, in the present work, the myrrh-treated group (G4) exhibited the highest IFN-γ levels (3.26 ± 1.22) with a delay in IL-10 production (0.92 ± 0.72). This delayed IL-10 expression in the infected myrrh-treated group allows IFN-γ to target newly born larvae, thereby reducing the number of larvae reaching circulation (4, 6). This result is consistent with previous research demonstrating elevated IFN-γ production in myrrh-treated mice early in infection, which may induce the immune system to initiate the inflammatory process and enable the host to control larvae production through enhanced cytotoxic killing by granulocytes, eosinophils, and activated macrophages (24, 25). While, in the group of infected animals treated with biosynthesized silver nanoparticles (G3), IFN-γ expression was observed earlier and at higher levels compared to the albendazole-treated group, suggesting that silver nanoparticles enhance the immune response at moderate level the immune response during the intestinal phase of *Trichinella* infection, unlike the Myrrh treated group. Conversely, groups treated with biosynthesized AgNPs and albendazole showed IL-10 expression peaking before the production of IFN-γ. This sequence limits the potential synergistic effect of anti-inflammatory cytokines. Taking into account, IL-10 is crucial for expelling *T. spiralis* adult worms from the small intestine by regulating the Th1/Th2 response at the intestinal mucosal surface (6, 37). These findings align with previous research that has shown chitosan administration to be associated with increased levels of IL-10 and an IgA response (38). A previous study (7) has highlighted the critical role of IL-10 in preventing hepatitis caused by the migration of intestinal T cells to the liver in mice infected with *T. spiralis*. Maintaining a balance between IFN-ɣ and IL-10 is crucial for regulating immunity against various stages of *T. spiralis* infection. Interestingly, the group treated with myrrh exhibited the highest immune response, characterized by early expression of IFN-ɣ and delayed expression of IL-10. This distinct pattern allows the immune system to swiftly eliminate and expel parasites, thereby enhancing overall immune. Due to limited resources, our study has several limitations, notably the inability to conduct a thorough evaluation encompassing all histopathological and immunological parameters discussed in our research over an extended period, spanning approximately 30 days. Instead, our assessment concentrated on the initial phases of infection and the host’s immune reaction up to the 6th day following infection. This limited duration constrained our ability to observe and analyze the entire process of migration and encystation of *T. spiralis* within muscle tissue. Nevertheless, despite this constraint, our results offer valuable insights into the early immune response during the intestinal phase of *T. spiralis* infection.

## 5 Conclusion

Considering the side effects associated with albendazole, the present study suggests that silver nanoparticles prepared with myrrh could be a promising alternative for treating the intestinal phase of trichinellosis. These nanoparticles demonstrated superior results compared to crude myrrh extract across all evaluation parameters, including effective parasite control in terms of percentage reductions, appropriate functional levels of IFN-γ and IL-10, and a significant reduction in MMP9 intensity compared to the positive control. Notably, the findings indicated that both silver nanoparticles prepared with myrrh and crude myrrh extract enhanced the immune response, suggesting that they might have complementary mechanisms of action against the parasites. Further investigations are advised to assess the histopathological and immunological parameters discussed in this study over an extended period, potentially up to 30 days. Additionally, the potential efficacy of using a combination of silver nanoparticles and myrrh to treat the muscular phase of trichinellosis should be explored.

## Data Availability

The original contributions presented in the study are included in the article/supplementary material, further inquiries can be directed to the corresponding author.
